# Growth of Large-Area, Stress-Free, and Bulk-Like 3C-SiC (100) Using 3C-SiC-on-Si in Vapor Phase Growth

**DOI:** 10.3390/ma12132179

**Published:** 2019-07-06

**Authors:** Philipp Schuh, Francesco La Via, Marco Mauceri, Marcin Zielinski, Peter J. Wellmann

**Affiliations:** 1 Crystal Growth Lab, Materials Department 6 (i-meet), FAU Erlangen-Nuremberg, Martensstr. 7, D-91058 Erlangen, Germany; 2 CNR-IMM, sezione di Catania, Stradale Primosole 50, I-95121 Catania, Italy; 3 LPE S.P.A., Sedicesima Strada, I-95121 Catania, Italy; 4 NOVASiC, Savoie Technolac, BP267, F-73375 Le Bourget-du-Lac Cedex, France

**Keywords:** 3C-SiC, PVT, sublimation sandwich, stress-free, bulk, large area

## Abstract

We report on the reproducible growth of two inch 3C-SiC crystals using the transfer of chemical vapor deposition (CVD)-grown (100) oriented epitaxial layers. Additional experiments, in which the diameter of the free-standing layers is increased, are presented, indicating the upscale potential of this process. The nucleation and growth of cubic silicon carbide is supported by XRD and Raman measurements. The rocking curve data yield a full-width-at-half-maximum (FWHM) between 138 to 140 arc sec for such grown material. Analysis of the inbuilt stress of the bulk-like material shows no indications of any residual stress.

## 1. Introduction

Silicon carbide is gaining more interest by the day, which leads to a better understanding of its growth when using physical vapor transport (PVT). Material grown with this technique reaches diameters of 200 mm and dislocation densities as low as 2800 cm^−1^ [1,2,3]. The cubic polytype, however, poses electrical advantages compared to the industrial available 4H- and 6H-SiC. With the smaller bandgap, near-interface traps created at the interface of SiC and SiO_2_ are located in the conduction band, increasing the charge mobility [4]. The method of consecutive growth and enlargement used for hexagonal polytypes (4H- and 6H-SiC) cannot be employed for the cubic (3C-SiC) one, as high supersaturation and temperature gradient are needed to stabilize this material. A method providing such process parameters is the sublimation “sandwich” method (SE) already presented by Tairov et al. [5] in 1976. Using such setup proved to be suitable to grow the 3C polytype [6,7]. A major drawback using any bulk growth method is the lack of a sufficient, high-quality seed. Such material can be obtained using the heteroepitaxial approach based on material growth on silicon substrates by chemical vapor deposition (CVD) [8,9,10]. However, due to the misfit between 3C-SiC and silicon, the wafers will feature a high stress levels leading to bended or even cracked material. Consequently, thickness presents limitations. By transferring CVD-grown layers on a SiC carrier, a seeding stack can be produced to achieve subsequent growth by SE [11]. Seeding material of high quality and a thickness close to 1 mm would allow enhanced bulk growth using methods like modified PVT (M-PVT) or continuous-feed PVT (CF-PVT), increasing the thickness even more [7,12,13].

Industrial relevance is obtained by increasing the total area of the transferred seeding layer and growing crystals on such large seeds. We present the reproducible growth of two inch material using this technique. Furthermore, preliminary experiments in a 100 mm setup are presented.

## 2. Experimental Methods 

**Figure 1:**
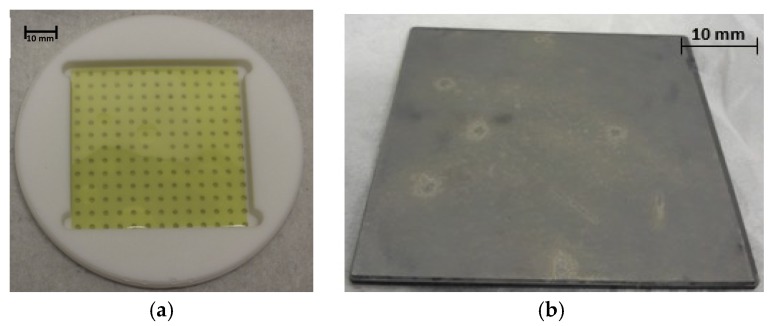
(**a**) Photography of a 52 × 52 mm^2^ free-standing cubic silicon carbide (3C-SiC) epitaxial layer after removing the silicon substrate. (**b**) Merged 3C-SiC-on-SiC seeding stack for a two inch process.

The used setup as well as numerical simulation data can be found in [11,14]. The manufacturing process, creating 3C-SiC-on-SiC seeding stacks, was done on four inch wafers featuring a thickness of approximately 20 µm for the epitaxial layer, on 580 µm-thick, highly n-doped, on-axis (100) silicon wafers. The sample size for two inch runs was reduced to 52 × 52 mm^2^ using a diode end-pumped solid-state laser (Rofin Power Line E20 LP, ND:YdO_4_, λ = 1064 nm), taking advantage of the multi-pulse ablation effect on 3C-SiC [15,16]. The resulting crack-free 3C-SiC-on-Si samples were removed from the silicon substrate using wet chemical etching in an HNA solution (hydrogen fluoride, nitric acid, and water (1:1:1.5)). As a result, free-standing epitaxial layers were then merged with a SiC carrier using a carbon glue layer whose main component was 1-methoxy-2-propanol acetate (Figure 1a,b).

Additional experiments were performed increasing the sample sizes, starting with a diameter of 95 mm or above by removing the overgrown edge areas of the wafers. On such seeding stack, subsequent sublimation growth was performed using a vapor phase setup with a sublimation “sandwich” cell. Three two inch and three four inch samples were manufactured with growth rates between approximately 160 µm/h and 320 µm/h and thicknesses between approximately 450 µm and 900 µm. For the removal of the resulting material from the SiC carrier, oxidation of the carbon glue layer at 800 °C and ultrasonic processing were performed. Characterization was conducted using Raman spectroscopy (Horiba Jobin LabRam HR Evolution confocal microscope, λ = 405 nm), X-ray diffraction (2θ-ω scans with analyzer), and optical microscopy.

## 3. Results and Discussion

**Figure 2:**
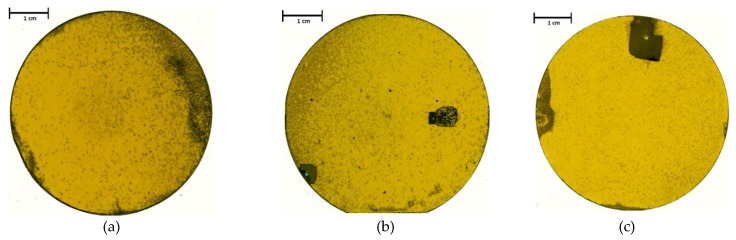
Through-Light optical scans of three free-standing 3C-SiC two inch wafers with an as-grown thickness of (**a**) 0.87 mm, (**b**) 0.78 mm, and (**c**) 0.5 mm. All three crystals were polished on both sides. Samples (**b**) and (**c**) feature burst areas.

The main challenge for this seeding layer transfer and bulk growth method was to obtain a crack-free epilayer large enough to fit in a two inch surface. The first attempts presented in [10] were carried out using a diamond wire saw, introducing mechanical force into the already stressed material. This method resulted in additional cracking along the (110) directions of the material. By utilizing the laser ablation process, it was possible to locally process the material and adjust the total dimensions of the used wafers. The resulting material was free of cracks, and therefore, an increased diameter for the sublimation epitaxial growth was achievable. Figure 2 shows the acquired crystals after removing the residual carrier and polishing (used grain sizes: 45–15 µm, 6–3 µm). The sample shown in (a) features a thickness of approximately 0.87 mm and a complete two inch diameter. The black spots in the center can be mainly assigned to protrusions increasing in size with increasing thickness. The black areas on the outer edge were generated by carbon contaminations on the seeding surface. The origin of such dirt was allocated to the merging step of epitaxial layer and carrier, overlapping at the edge of the seeds. The sample shown in (b) features less inhomogeneities at the edge but presents two bigger areas featuring a burst of the seed. This effect was due to an enclosure of gases between the seed and the carrier, leading to the local removal of the material in such areas during vapor growth. Increasing the temperature would increase the pressure of the gas inclusions, inevitably bursting the epitaxial surface which presented the weakest escape route. The sample shown in Figure 2c exhibits similar burst effects in the top area and contaminations on the three edge areas. The main defect on all samples, however, was caused by the protrusion generated in the carbonization step during the CVD growth of the seeding layer.

**Figure 3:**
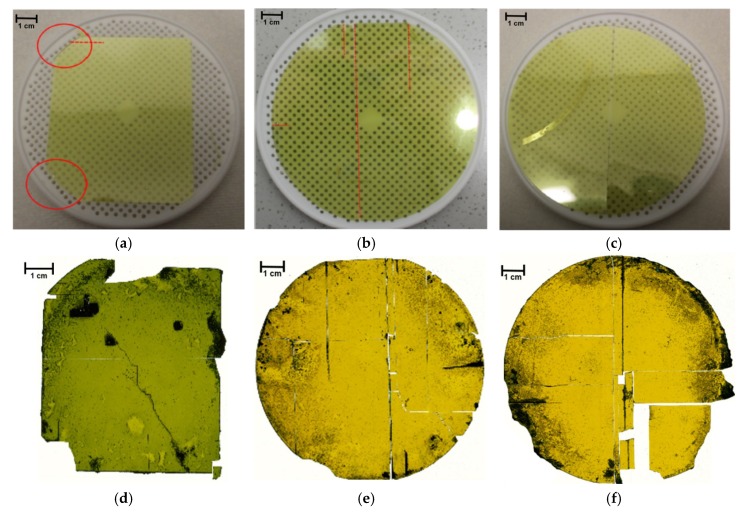
Free-standing epitaxial layers cut out of a four inch wafer increasing the diameter of the grown samples. (**a**) Features of some edge areas of the wafer at the corners circled in red. Cracks started to propagate from such edge areas. (**b**) The epitaxial layer was removed from the edge areas, and cracks propagated on the flat areas. (**c**) Reduction to a 95 mm diameter without edge or flat areas resulted in a single crack generated by uplifting during etching. The corresponding crystals grown on the shown free-standing epitaxial layers are depicted in (**d**–**f**). All samples feature additional cracks introduced by the post-process removal from the carrier due to polycrystalline growth at the borders. The thicknesses of the samples varied between 480 µm to 520 µm, with growth rates between 180 µm/h and 320 µm/h.

Increasing the diameter of the epitaxial layer augmented the cracking probability during the etching but not during the cutting. Figure 3a–c shows three free-standing 20 µm-thick epitaxial layers with various cracks, appearing during the substrate removal step. The breakage visible in Figure 3a started to propagate at the edge areas highlighted by red circles. The material in such region featured inhomogeneities involving the CVD process, overgrowing the border of the silicon substrate. This led to a predetermined breaking point. The sample shown in Figure 3b shows a layer without any residuals of this border area. To maximize the area output, flats were included. The cracks appearing in this experiment started from the flats. Therefore, an additional laser ablation run reduced the total diameter to 95 mm, as shown in Figure 3c. Only one crack appeared in the middle of the sample, caused by the uplift of the layer during etching. The chemical removal of the substrate initiated at the edges, moving into the center. Because of gas bubbles adhering to the silicon, an uplift of the epitaxial layer occurred, tilting it in the center. This tilt led to breakage by mechanical stress. Even though the layers broke during substrate removal, a merging step with a SiC carrier was performed. The resulting stacks were implemented into an adapted four inch growth cell, enabling the growth of larger diameter material. The manufactured crystals can be seen in Figure 3d–f. The depicted samples varied in thickness from 480 µm up to 520 µm, and their growth rates varied from 180 µm/h up to 320 µm/h. Additional cracking of the material occurred mainly during the removal of the grown material from the residual SiC carrier. For the growth of such diameters, a spacer with a 100 mm hole was used. As the utilized seeding layers exhibited a smaller dimension of ca. 95 mm and, additionally, were cracked during the prior seed preparation step, a seedless growth partially occurred on the carrier. The material grown on such seedless regions nucleated spontaneously as polycrystalline material. This unwanted growth clamped the material grown on the actual seeding layer. For the final removal of the resulting crystal from the carrier, an additional mechanical force was thus needed. Therefore, additional breakage at this point was inevitable. Apart from the cracks, all samples featured similar defects which are also visible on the two inch material. All crystals grown by this method featured the typical yellow appearance of cubic silicon carbide. Rocking curve measurements of the 002 reflex resulted in a full-width-at-half-maximum (FWHM) around 138 to 140 arc sec. These values are almost comparable with those of high-quality material grown on hexagonal SiC substrates, which can be lower than 120 arc sec [6].

**Figure 4:**
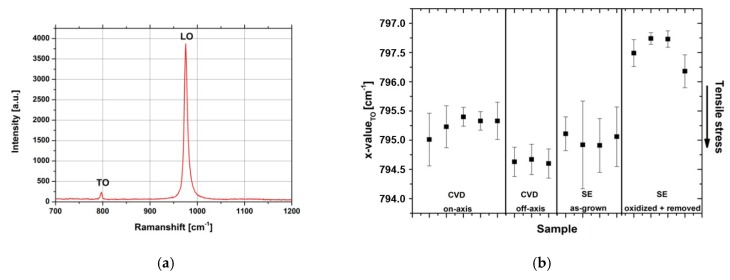
(**a**) Typical Raman spectrum of SE-grown material in a protrusion- and defect-free surface area. (**b**) Comparison of the x-value for the transversal optical (TO) mode of 3C-SiC epitaxial layers grown by chemical vapor deposition (CVD) (on and 4° off-axis), homoepitaxial as-grown material using the SE setup, and after oxidation and removal from the carrier. The obtained values feature a trend of stress reduction for the final material.

Raman analysis of 3C-SiC-on-Si, as-grown 3C-SiC-on-SiC, and post-processed SE-grown material was performed. Figure 4a shows a resembling spectrum of a defect-free surface area on SE-grown material. The transversal optical (TO) mode is forbidden for defect-free (100) on-axis material. However, the stress in the material can be determined from the peak position [17]. The plot in Figure 4b shows the wavenumber of the TO mode for typically stressed CVD-grown material on on-axis and 4° off-axis silicon substrates. From this data, a tensile stress was visible in both materials. In comparison, as-grown material grown by SE featured similar values as CVD-grown material on on-axis substrates. However, after the oxidation and removal of the crystal from the carrier, the material tended to reduce the stress in the direction of 797 cm^−1^. 

Additional XRD 2θ-ω scans were performed on various samples after the removal from the carrier grown by SE. From these data, the actual lattice parameters of the material were calculated. Table 1 shows the resulting values. The *ε* value describes the distortion by subtracting both values and dividing by the in-plane value. A distortion below 10^−5^ corresponds to the experimental error of the used setup and supports the assumption of stress-free material.

## 4. Conclusions

We presented a method to reproducibly manufacture free-standing epitaxial layers up to 52 × 52 mm^2^ using a laser ablation process. For increased dimensions, cracking occurred during wet-chemical etching. The resulting epitaxial layers were repeatedly merged with SiC carriers. The resulting seeding stacks were then applied for the growth of two and four inch material using a high-temperature vapor growth setup. All samples grown by this method exhibited a thickness between 320 µm and 520 µm and grew at rates between 190 µm/h and 320 µm/h. The optical appearance of all crystals featured a bright yellow color, typical for cubic silicon carbide. XRD analysis and Raman spectroscopy confirmed 3C-SiC growth. The typical defects of (100) 3C-SiC, such as Stacking faults, Anti-Phase-Boundaries, and protrusions were present. Mainly, surface contaminations on the seed altered the material quality, indicating the need of a cleaning process prior to the growth. XRD and Raman analysis of the bulk material grown with this method proved the growth of stress-free material.

## Figures and Tables

**Table 1:** Lattice parameters calculated from XRD 2θ-ω scans on SE-grown material and 3C-SiC-on-Si material.

Sample	Orientation	*a* ‖ *(Å)*	*a* ⊥ *(Å)*	*ε*
SE123	On-axis	4.3599	4.3602	−7.9 × 10^−5^
SE126	On-axis	4.3617	4.3608	2.1 × 10^−4^
3C-SiC-on-Si	On-axis	4.3628	4.3582	1.1 × 10^−3^
SE136	4° off	4.3602	4.3602	−3.7 × 10^−6^
SE137	4° off	4.3605	4.3602	7.1 × 10^−5^
3C-SiC-on-Si	4° off	4.3633	4.3582	1.2 × 10^−3^
